# Clinical Congenital Anophthalmos and Microphthalmos—Experiences of Patients and Their Parents after More than 10 Years of Treatment

**DOI:** 10.3390/children10010034

**Published:** 2022-12-24

**Authors:** Stefanie Frech, Markus Schulze Schwering, Michael P. Schittkowski, Rudolf F. Guthoff

**Affiliations:** 1 Department of Ophthalmology, Rostock University Medical Center, 18057 Rostock, Germany; 2 Medical Center Echternach, Ophthalmology, 6463 Echternach, Luxembourg; 3 Department of Ophthalmology, Division of Strabology, Neuroophthalmology and Oculoplastic Surgery, University Medical Center Göttingen, 37075 Göttingen, Germany

**Keywords:** congenital clinical anophthalmos, blind microphthalmos, ocular prothesis, questionnaire, social and emotional stability

## Abstract

Congenital clinical anophthalmos and blind microphthalmos describe the absence of an eye or the presence of a small eye in the orbit. Between 1999 and 2013, 97 children with anophthalmos or microphthalmos were treated with self-inflating, hydrophilic gel expanders at the Rostock Eye Clinic. More than a decade later, this study investigated the perspective of patients and parents regarding the treatment, the surgical outcome, and the emotional and social well-being of the patients. A total of 22 families with 16 patients sighted in the other eye and six patients blind in both eyes participated. Questionnaires were developed, including items on physical, emotional, social, and medical aspects. The patients felt emotionally stable and integrated into their social environment, with no major limitations reported by the majority. These statements were confirmed by most of the parents. Parents (67%) indicated that the success of the operation was already apparent after the first intervention and that the current situation did not play a role in the patients’ social environment. The study provided new insights into the therapy results, the postoperative care, and the social and emotional stability of the prosthesis-wearing patients, indicating the chosen expander methods as promising in terms of positive postoperative care.

## 1. Introduction

Congenital anophthalmos is a rare ocular anomaly in which histologically detectable eyeball structures are absent due to poor development of the primary optic nerve vesicle [1,2]. However, with appropriate imaging or intraoperatively, rudimentary remains of this structure can usually be detected [3]. Therefore, the term clinical congenital anophthalmos seems more appropriate and denotes a phenotypic range between microphthalmos and anophthalmos [4]. Functionless blind microphthalmos is characterised by a reduced volume of the eyeball with possibly hypoplastic lid and conjunctival structures [3].

Both clinical pictures lead to visible abnormalities that can be associated with psychosocial difficulties. In bilateral manifestations, there is blindness [5]. Unilateral and bilateral findings may be isolated or associated with a variety of abnormalities and syndromes [6]. The diseases are caused by disorders in the morphogenesis of the eye [7]. These can be caused by genetic defects, such as the presence of chromosomal aberrations or monogenetic mutations [8], or by external influences that affect the processes of morphogenesis, such as intrauterine viral or toxoplasmosis infection or exposure to toxins during pregnancy [9]. Described prevalence rates for anophthalmos/microphthalmos vary from 1.2 to 3.21 per 10,000 live births [10,11,12,13,14,15,16]. Thus, the diseases belong to the group of rare diseases defined by a prevalence of <5/10,000 in Europe [17].

Therapy with the goal of prosthesis capability includes various aspects that must be taken into account, such as widening of the eyelid fissure, enlargement of the eye socket, as well as the shape of the prosthesis socket and the orbital volume [3,5]. In the context of the development and establishment of new treatment strategies, including self-inflating highly hydrophilic hydrogel expanders, 97 patients were treated at the Department of Ophthalmology at the Rostock University Medical Centre in the years 1999–2013 [3,6,18,19,20]. The therapy of congenital clinical anophthalmos as well as blind microphthalmos requires a multidisciplinary approach including paediatricians, ophthalmologists, oral and maxillofacial surgeons, and human geneticists. The children and their families face intensive medical and surgical treatment, including numerous hospitalisations, often with surgical procedures, and intensive care by ocularists. In addition, families are confronted with uncertainty about the future situation of their outwardly striking child, who may be blind, and they face particular challenges with regard to school, education, and employment, as well as the child’s independence. This is especially true, as associated pathologies or systemic abnormalities can also affect lifestyle and family life. Little has been described about the effects on the reactions of the mainly functional rehabilitation of the eye socket in the absence of an eyeball, social integrity, and health-related quality of life (QoL) [21,22].

The aim of our study was to examine the therapy measures carried out, treatment results, and current living conditions after more than a decade of treatment initiation from the perspective of parents and the patients who are now in their teens and young adulthood.

## 2. Materials and Methods

### 2.1. Development of an Anophthalmic/Microphthalmic Questionnaire for Parents and Patients

One questionnaire for parents and one for the patients (children) were newly generated in order to shed light on the perspectives of both generations. Topics included physical, emotional, social and medical aspects of the diseases. Furthermore, the questionnaires were specifically adapted to sighted and blind patients and parents of sighted and blind patients, so a total of 4 questionnaires were established. The children’s questionnaire included a total of 18 questions in the above-mentioned 4 topics for the sighted children and 17 questions for the blind children; the parents’ questionnaire included 5 dimensions with a total of 28 questions. Furthermore, the participating patients were asked to describe their dreams and career aspirations in an open question field.

**Figure 1:**
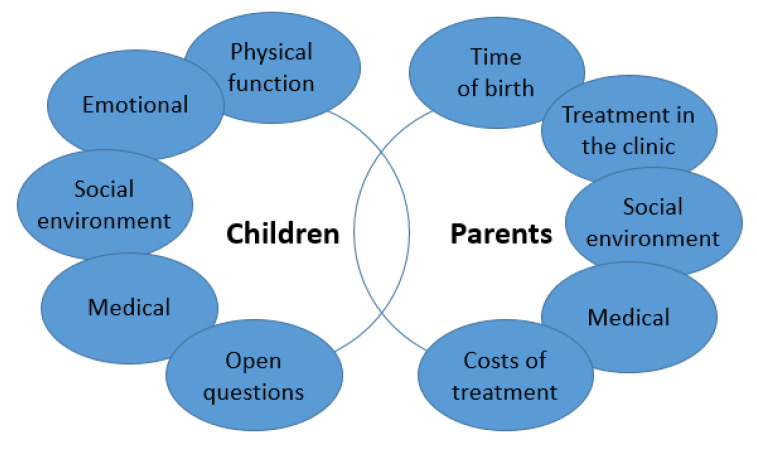
Dimensions of the newly developed questionnaire for children and parents.

Figure 1 summarises the dimensions of the questionnaires. Parents and their children were asked to answer the questions using a 3-point scale (yes, to some extent, no). Unanswered or ambiguous questions were excluded from the analysis. The results are presented as percentages in the continuous text. In the tables, the number of respective answers was indicated. The content of the open-ended questions was classified and evaluated using a category system and supported by quotations.

### 2.2. Study Design and Study Population

Of the 97 patients who had received treatment in Rostock, a request for participation with information about the study was sent to 86 families (parents and patients), more than a decade after the first intervention. Contact details were missing for 11 families. Patients (47 female, 39 male) were born with unilateral congenital clinical anophthalmos or blind microphthalmos and had received one or more treatments in Rostock between 1999 and 2013. In addition to congenital anophthalmos and blind microphthalmos, other pathologies and/or systemic anomalies might have been associated. In 2009, Schittkowski and Guthoff investigated the frequencies of systemic diseases and identified possible pathologies [6].

**Figure 2:**
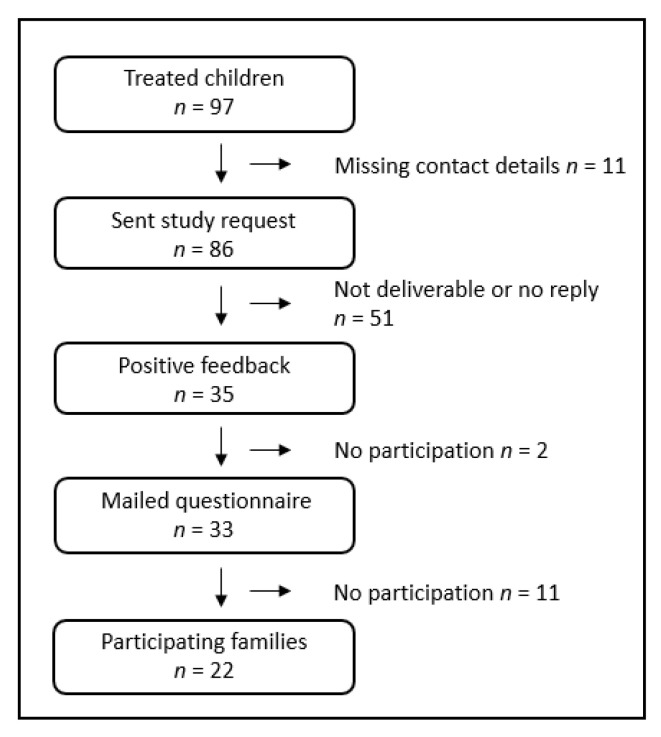
Recruitment of study participants.

A positive response came from 35 families, with 33 agreeing to participate in the study. The questionnaires were sent to these families. In total, 22 families participated in the survey, which corresponded to a response rate of 25.6% of the study requests sent out and a response rate of 62.9% of those who agreed to participate. The patients with unilateral anophthalmos or microphthalmos (12 + 4) were sent the questionnaire for “sighted children”. Two blind patients with unilateral anophthalmos and blind partner eye and four patients with bilateral anophthalmos filled in the questionnaire for “blind children”, which was adapted in some respects to the non-existing visual ability. The questionnaires were completed by 21 parents and 19 patients. The recruitment of the study participants is shown in Figure 2.

## 3. Results

### 3.1. Study Population

Of the patients, 45.5% were female and 54.5% were male. At the time of the study, they were on average 15.7 years old (from 8 to 23 years). Among them, 14 had a diagnosis of unilateral clinical anophthalmos, 4 unilateral microphthalmos and 4 bilateral clinical anophthalmos. Of the 14 patients with unilateral clinical anophthalmos, 14 had a second healthy eye and 2 were also blind in the second eye, resulting in 6 patients (27%) being blind in both eyes and 16 patients with a healthy second eye.

**Figure 3:**
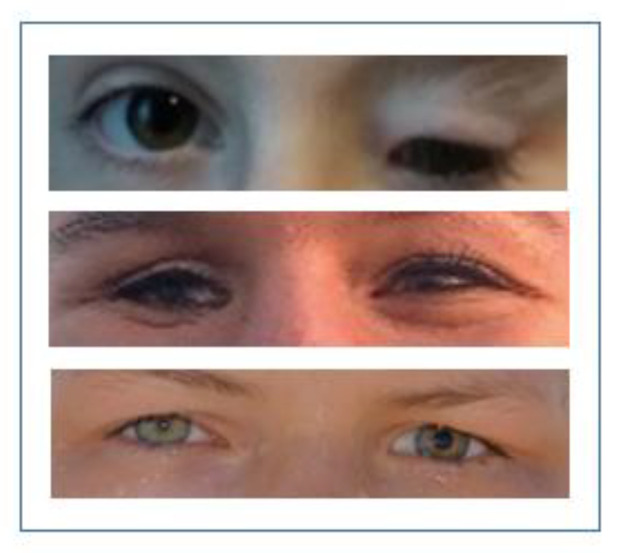
Patients after more than 10 years of treatment or monitoring by doctors and ocularists. Top: anophthalmos (left), middle: anophthalmos (right), bottom: microphthalmos (left).

Table 1 presents an overview of the study population, while in Figure 3, selected patient examples are shown.

### 3.2. Unilateral Clinical Anophthalmos and Blind Microphthalmos with Sighted Eye

The questionnaire was completed by 15 participating teenagers and young adults (Table 2). The majority of patients (73%) did not feel noticeably physically limited by seeing with only one eye. When looking in the mirror, half did not notice much difference between the prosthesis and the sighted eye. For 73%, there were no problems with vision at school or at the training place.

Seeing with only one eye did not cause any negative emotions for most of them, such as anger (80%), fear (87%), anxiety (73%) or sadness (67%). Moreover, 80% said they had gotten used to the conditions, but almost half of the respondents (47%) were afraid that their remaining eyesight might weaken in the future.

In terms of social environment, 71% of patients reported playing just like other sighted children, and 7% had contact with others who wore a prosthetic eye. In 40% of the patients, the malformation of the eye was not a frequent topic of conversation, although 40% reported that others noticed the malformation and asked them about their appearance. However, this factor did not cause 80% to be afraid of making new contacts, and they felt accepted as they are (79%).

For 83% of the study participants, the fit of the prosthesis did not need to be corrected more than once a year. Moreover, 87% were not afraid of visits to the ocularist or ophthalmologist and 13% had to visit the ophthalmologist more than twice a year. Additionally, 36% of the participants reported other conditions besides congenital clinical anophthalmos or blind microphthalmos.

### 3.3. Bilateral Clinical Anophthalmos

The questionnaire was completed by four participating teenagers and young adults (Table 3). Half stayed only in familiar places and 75% were dependent on personal help. One patient said they had been taught all the skills at school to be able to act independently. Devices used were a stick for the blind (*n* = 3) and a mobile phone with voice output (*n* = 1).

Emotions triggered by blindness were sadness (25%), worry (25%) and fear (25%). Three-quarters of the patients were used to the situation, and 25% were afraid of not being able to cope on their own.

All patients felt accepted in their social environment as they are and no one had any fear of meeting new people. Half said they were sometimes confronted with their appearance and 25% were not aware of their eye malformation. For 50%, the malformation of the eye was not a frequent topic of conversation and 50% had contact with other people with one or two prosthetic eyes.

The prostheses did not cause any pain in any of the patients, and the question about the fear of visiting the ophthalmologist or ocularist was answered negatively by all of them. A quarter had to visit the ophthalmologist more than twice a year, and 50% had to have their prosthesis adjusted more than once a year.

### 3.4. Parental Statements of Patients with Unilateral and Bilateral Clinical Anophthalmos or Blind Microphthalmos

#### 3.4.1. Around the Time of Birth

During the ultrasound examination in pregnancy, no malformation of the child was detected in 93% of cases with unilateral findings and in 67% of cases with bilateral findings. Slightly more than half (53%) of parents with monolateral findings and 50% of parents with bilateral findings were affected when they saw their child for the first time, with the doctor only being able to explain what the malformation was in 20% of the monolateral and 17% of the bilateral cases. In order to understand what kind of condition their child had, 53% of the parents with monolateral findings and 25% of the parents with bilateral findings consulted books and the internet.

#### 3.4.2. Treatment at the University Eye Hospital Rostock

From the families with monolateral congenital clinical anophthalmos or blind microphthalmos, 14% were referred to the Eye Clinic Rostock by their treating doctor, and 60% became aware of the clinic through their own research. Of the bilateral diagnoses, 67% were referred to the Eye Clinic Rostock, and 33% had found the clinic through their own research. Once seen, the team of doctors, nurses and ocularists addressed the questions, fears and concerns in 80% of the families with monolateral findings and 73% were able to recognise surgical success after the first procedure. For bilateral findings, all families reported that the clinic team addressed their questions, concerns and fears. In 50% of the children, success was evident after the first surgical intervention, and in 50% of the children, partial success was visible.

#### 3.4.3. Costs of Surgical and Conservative Treatments

For 47% of the families with monolateral congenital clinical anophthalmos or blind microphthalmos, all costs such as travel, hospital stays and operations were completely covered by the statutory health insurance or private insurance. An own cost share of more than 35% of the treatment costs was experienced by 20%, in contrast to 67% who had no worries about new costs in the course of further treatments. For bilateral cases, 83% of families had all costs covered by health insurance and 30% were worried about new costs in the course of further treatment. In addition to monolateral clinical anophthalmos or microphthalmos, 30% of the patients had other health limitations according to the parents, and 67% of the patients with bilateral clinical anophthalmos.

#### 3.4.4. Social Environment of Patients

All parents stated that the patients with monolateral congenital clinical anophthalmos or blind microphthalmos were doing fine in kindergarten. At school, the proportion of positive responses decreased to 67%. Nearly half of the patients (47%) played like other children, and 64% were not afraid to make new contacts because of their appearance. In the bilateral findings, the question about the patients getting along well in kindergarten and school was answered yes by 50% of the parents. One-third of the blind patients played mainly with other blind children, and according to the parents, 83% of the patients were afraid of meeting other children or making new contacts because of their appearance.

The look of the child with monolateral findings was addressed by 29% of the parents, whereby the malformation was not noticeable at first sight according to 20%. Of the parents with blind children, 40% were asked about the appearance of the child, with half of the parents saying that the malformation was not noticeable at first sight.

In 43% of the families, the unilateral malformation of the eye was a topic of conversation between the parents and between the parents and their child. It was not a topic with other parents in 64% of the families. The bilateral malformation was a topic of conversation between the parents in 20%, and in 60% of the cases also between the parents and the affected child. In 33% of the cases, the malformation was discussed with other parents.

Within the social environment, monocular vision played no role in 73% of the families and bilateral blindness of the child no role in 50% of the families. Positive answers regarding their children’s career plans were given by 89% of the parents of children with sighted partner eyes and 17% of the parents of a blind patient. No parents of a child with unilateral congenital clinical anophthalmos or blind microphthalmos exchanged information via a self-help group, although 20% had contact with other families in the same situation. In contrast, half of the parents of a blind patient were in private contact with others in the same situation, 17% through a self-help group.

Table 4 shows the parents’ responses, subdivided according to dimensions and mono- or bilateral blindness of the children.

### 3.5. Open Question Section of Unilateral and Bilateral Patients with Clinical Anophthalmos or Blind Microphthalmos

**Figure 4:**
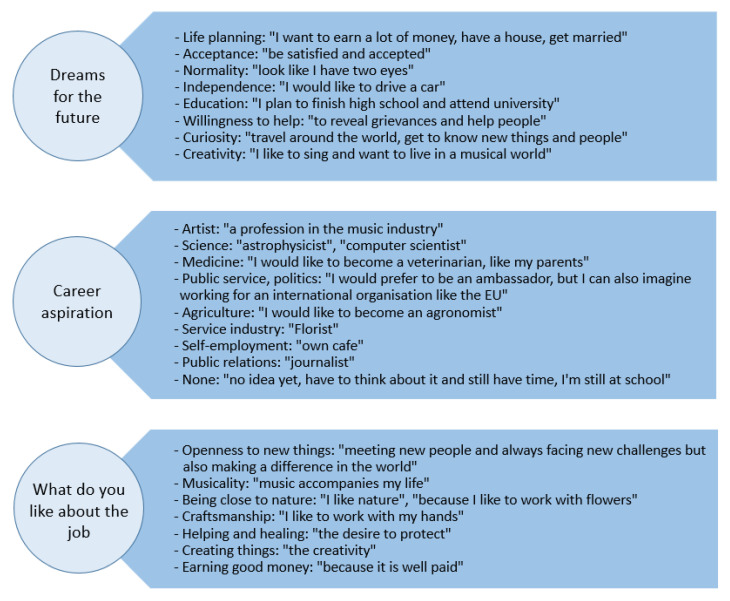
Summary of the open question section. Answers were divided into categories and supported by quotations (in quotation marks).

Figure 4 shows the results for the open-ended questions regarding patients’ career plans and their dreams. Dreams were expressed in diverse directions that included life planning, society, education and personal aspects. Career plans and ideas about working life were described in great detail. Although there is a limitation in career choice due to at least one blind eye, the children expressed career aspirations and dreams in many different occupational fields.

## 4. Discussion

For most patients, after more than a decade, the families treated at the Rostock Eye Clinic were contacted with the aim of recording and analysing the therapy measures carried out at that time, the treatment results as well as the current living conditions with a developed questionnaire. The lack of an anatomical eye structure and the associated complete or partial loss of vision is a great challenge for the patients and their parents, which can influence their self-esteem, personal relationships or even their social environment. The questionnaire allowed insight into the family and social structures and examined emotional and medical aspects of the treatment.

### 4.1. Prenatal Diagnosis, Birth and Initial Treatment in the Eye Clinic Rostock

In Germany, ultrasound diagnostics are regularly provided during pregnancy from weeks 11 to 14 with regard to organ screening, in which gross malformations, e.g., of the heart or brain, are detected [23]. If there is a high-risk pregnancy, extended malformation diagnostics are advised, which diagnoses more specific malformations [24]. In this study, in 14 of the 15 families with monolateral findings and in four of the six families with bilateral findings, no physical change or malformation of the child was detected during the ultrasound examination. Thus, it can be assumed that there was no indication for an extended diagnosis of malformation. For this reason, the question remains open whether an abortion for maternal reasons would have been considered if the malformation had been known, as described in other case reports [25,26]. The prenatal diagnosis of a disease plays an important role in the parents’ decision between continuation of the pregnancy and an abortion, but also enables psychological preparation and planning in dealing with the disease after birth.

At the Eye Clinic Rostock (Table 4, “Treatment at the University Eye Hospital”), the ophthalmologist discusses the condition with the parents and offers a treatment plan. Parents are understandably concerned about the condition, especially if the finding was not diagnosed prenatally [7]. This is also shown by the results of our study, in which 11 out of 21 parents were affected when they saw their child for the first time. The condition may also have further-reaching family consequences, as in discussions with parents, the difficulty of accepting the child with its aesthetically inadequately corrected disability was also expressed [3]. The discussions about the diagnosis, the aetiology of the disease and the treatment in the eye clinic were important. According to 18 parents, the team of the clinic responded to the questions, fears and concerns, especially as prior to the treatment in the eye clinic, the treating doctors could not explain the change to a large extent. Therefore, an efficient multidisciplinary team is important for a positive further development of the young patients, but also for the support and advice of their parents.

### 4.2. Social Environment and Emotional Aspects of Life with Ocular Prosthesis in Young Patients with Congenital Clinical Anophthalmos or Blind Microphthalmos

The majority of the young patients were well integrated into their social environment and they felt accepted. Seeing with only one eye caused just a few negative emotions, and 80% got used to the situation and were not afraid to make new contacts. This statement was also made by all blind patients. The results imply a stable environment in which the children grew up and had learned to cope with their situation of anophthalmia, which they were also confronted with. They felt like full members of their communities despite the prosthesis. That is why the results also illustrate that the surgical intervention in early childhood and the postoperative care including family integration may have contributed to the positive outcome of our study.

An important aspect here is the young age at which the surgical intervention had taken place, because the degree of disability and the age of the patient can influence the individual acceptance of the anophthalmos [27,28]. The contribution of the expression of visual disability was also reported by Avery and Hardy, 2014. Children with two visually impaired eyes showed greater social difficulties compared to children with only one eye affected [29]. Not only did the patients naturally have difficulties in performing activities of daily living, but the condition also had a negative impact on their participation and enjoyment of social activities. This is in contrast to the findings in this study where all blind patients felt socially integrated.

The impact of congenital clinical anophthalmos or blind microphthalmos on the quality of life of young patients has been sparsely investigated. Two studies addressed this issue, and both included parents’ perceptions [21,22]. Casslén and colleagues assessed health-related quality of life (HR-QoL) and vision-related quality of life (VR-QoL) in 15 individuals with unilateral anophthalmos and microphthalmos aged 1.7 to 14.1 years who were treated with ocular prosthesis [21]. The results showed that children and adolescents with congenital clinical anophthalmos or blind microphthalmos had a poorer quality of life than healthy individuals, which was also reported by parents, and the condition had a negative impact on psychosocial well-being [21]. Similar reduced scores have been reported in other quality of life studies of children and adolescents with various ophthalmic diagnoses (cataract, glaucoma) on self-rated and parent-rated quality of life [30,31,32,33]. In 2018, Dahlman-Nohr and colleagues determined child and parental perceptions of functional visual ability (FVA) and vision- and health-related quality of life (VR-QoL, HR-QoL) in 45 children with microphthalmia/anophthalmia/coloboma (MAC) aged 2 to 16 years. Both quality of life parameters were significantly reduced, with parents rating the condition as less important for HR-QoL than the children themselves [22].

Consequently, it is important to thoroughly examine every congenital clinical anophthalmos or blind microphthalmos child and initiate treatment, including provision of an ocular prosthesis. An examination of the other eye is necessary to detect possible visual impairment at an early stage [21]. The importance of the still-sighted eye for the patient’s emotional well-being was also evident from the results of our study, which asked about the fear that the eyesight might decrease in the future (Table 2 and Table 3, “Emotional”). The loss of the second eye was a topic that triggered fear in almost 50% of the patients. Vision loss that could spread to the other eye was also associated with fear and anxiety in other studies of anophthalmic adult patients who received a prosthesis [34,35]. The Brazilian patients in the study by Goiato et al. were also depressed after losing the eye. It was only after a while that the loss and the current situation were accepted and a social reintegration took place with the help of the prosthesis and the changed appearance [34]. This aspect was different in our study. The children were surgically treated at a very early stage of their lives, so they had never known their situation and appearance differently. Rokohl and colleagues identified the most common concerns and their changes over time, also with respect to different parameters such as age, gender or reason for eye loss, in 106 patients. The health of the remaining eye was the most common concern, necessitating the need for good ophthalmic follow-up [36]. Concerns about the health of the other eye were expressed by 65% of patients in the study by Ruiters et al. In this study, a negative impact of a prosthesis on social relationships had not been shown [37]. Therefore, it is important to assess both the physical and emotional well-being of anophthalmic patients in order to identify those patients who need additional physical and mental support [38].

### 4.3. Factors That Positively and Negatively Influence the Well-Being of Anophthalmic Patients

Makrakis and colleagues evaluated quality of life, stress and individual anxiety after enucleation and investigated adaptation difficulties after receiving ocular prostheses. The most common complaints included depression, anxiety, altered personality and health perceptions, negative socioeconomic influences and feelings of insecurity and social rejection [27]. Wang and colleagues analysed in their study the psychological symptoms and associated factors in 150 anophthalmic patients with ocular prostheses. The patients showed increased levels of somatisation, depression and anxiety compared to control persons. Furthermore, the severity of psychological symptoms in patients was associated with gender, age, marital status, level of education and cause of enucleation [28]. Thus, prosthetic restoration played an important role in improving the patients’ personal situation and reintegration into society [39]. In contrast, only very few negative emotions such as anger, fear, sadness or anxiety were reported by the patients in our study, which could again be a consequence of the early surgical intervention, pushing these negative emotions into the background due to good social integration. The importance of a prosthesis in contributing to patient well-being was also demonstrated in a study of 36 patients before and after the provision of an ocular prosthesis. The treatment led to a significant improvement in psychosocial problems and other variables studied, such as anxiety and depression [40]. Moreover, in the recent study by Ruiters et al., adult patients reported satisfaction with the physical appearance of the artificial eye and adequate psychosocial integration [37].

### 4.4. Medical Aspects of Ocular Protheses

Another dimension of the questionnaire was related to medical aspects of the prostheses (Table 2 and Table 3, “Medical”). In 80% of the patients, there were no complications with the postoperative treatment and the prosthesis with regular annual check-ups. These results imply that, on the one hand, the early surgical intervention provided a good basis for further postoperative follow-up and treatment, which was only associated with pain in rare cases. On the other hand, they pointed to professional training and education in the use of the prosthesis with regard to cleaning and handling. Contradictory results were described in the study by Song et al., in which the majority of the participants had difficulties in handling the cleaning of the prosthesis, which the authors associated with an educational deficit of the medical staff [41].

### 4.5. Self-Perception and Perception by Others

Another aspect of the present study was the question of how the young patients perceive their external appearance and how they are perceived by others (Table 2, Table 3 and Table 4, “Social Environment”). The surgical procedure and the wearing of a prosthesis have a great influence on the patients’ self-image and their quality of life [36,42]. Although individual patient stories vary greatly, all patients hope that their appearance after surgery is positively improved in the sense of a natural appearance that reflects a natural looking eye.

Regarding their appearance, the patients’ reactions in our study were interesting. Half of them said they did not see much difference between the prosthesis and the eye in the mirror. It should be taken into consideration that the patients never perceived themselves differently either, as they had been familiar with the prosthesis all their lives and had also become accustomed to it (80%). One female teenager told us that she had removed the prosthesis when she was 15 years old because she preferred a natural appearance. Less than half answered “yes” to the question, “I am often asked about my appearance”. The results somewhat mirror those of Song and colleagues [41]. They studied the satisfaction of 78 patients aged 7 to 74 years with a prosthesis and determined the variables that were related to it with the aim of identifying those that increase satisfaction. These variables included economic status and the reactions of other people. Almost half (48.7%) of their patients reported that other people hardly recognised that they wore a prosthesis, 46.2% reported that others partly recognised and 5.1% that others easily recognised the prosthesis [41]. A better understanding of the determinants of patient satisfaction may therefore provide useful information for improving treatment.

The unilateral and bilateral blindness inevitably leads to limitations in career choices. The results of the open-ended questions (Figure 4) in this study showed career aspirations in a wide range of areas that require creativity and knowledge. No occupational wishes were expressed that could not be carried out due to the visual impairment. Either these professions were directly excluded because the patients knew that they could not take up certain professions or they thought less in a three-dimensional world and thus directly excluded certain professions.

### 4.6. Strengths and Limitations of the Study

A strength of the study was the joint presentation of the views of parents and children, so that the care process was illuminated from different perspectives. The size of the study conducted was primarily exploratory. It is likely that only those families who had a positive attitude towards the treatment and a great interest in the care of their child gave feedback and participated further. Therefore, it can be assumed that the actual care situation could be worse than represented here in the present study. The long period of more than 10 years (except for two patients, who were 8 years old when the study was performed) between the surgical intervention and this study means that only limited patient data were available. This was also due to the fact that some of the patients from abroad, who often travelled very long distances, only presented once and received further treatment at their place of residence. It is therefore not possible to estimate which costs in detail were not reimbursed and which other financial (lack of health care) and structural problems (access to ocularist treatment) might play a role in the overall assessment of the treatment outcome.

## 5. Conclusions

The patients participating in this study felt emotionally stable after treatment of their clinical congenital anophthalmos or blind microphthalmos more than a decade ago. They were integrated into their social environment, and the majority reported no noticeable limitations. These statements were confirmed by the majority of their parents. The results confirm the approach of the surgical measures carried out at that time and the therapeutic results of the prosthesis treatment, which created a remarkable outcome in terms of self-perception, perception of others and social integration, and provided new insights into the social and emotional stability of the patients today.

## Figures and Tables

**Table 1:** Classification of the study population with regard to their disease.

	Unilateral Congenital Clinical Anophthalmos	Bilateral Congenital Clinical Anophthalmos	Unilateral Blind Microphthalmos	Total (%)
Male	7	2	3	12 (54)
Female	7	2	1	10 (46)
Total	14	4	4	22 (100)

**Table 2:** Frequencies of responses of patients with unilateral clinical anophthalmos and blind microphthalmos with healthy second eye.

Physical Function	Yes	To Some Extent	No
I don’t feel restricted by seeing with just one eye	11	3	1
When I look in the mirror, I don’t see a big difference between the prothesis and my eye	4	3	7
I see everything I need in the booklet and on the blackboard at school/at the training place	11	2	2
Emotional	Yes	To Some Extent	No
The fact that I can only see with one eye makes me:			
Sad	2	3	10
Angry	1	2	12
Worried	3	1	11
Afraid or anxious	1	1	13
I got used to it	12	3	0
I am afraid that my only eyesight could fade in the future	7	4	4
Social Environment	Yes	To Some Extent	No
I feel/felt accepted as I am	11	3	0
I’m often asked about my appearance	2	7	5
I am playing/played just like other seeing children	10	3	1
I’m afraid of making new contacts because of my blindness	1	2	12
Other people don’t even notice the malformation of the eye	2	7	6
The malformation of the eye is a frequent topic of conversation	1	8	6
I have contact with other children who also wear one or two eye prostheses	1	1	13
Medical	Yes	To Some Extent	No
I have to see an ophthalmologist more than twice a year	2	0	13
I am afraid of visiting an ophthalmologist or ocularist	1	1	13
The side with the prosthesis often hurts	0	0	0
The side with the prosthesis sometimes hurts	6	0	0
The side with the prosthesis never hurts	8	0	0
The position of the prostheses must be corrected more than once a year	2	0	10
I have other diseases	5	0	9

**Table 3:** Frequencies of responses of patients with bilateral clinical anophthalmos.

Physical Function	Yes	To Some Extent	No
My blindness restricts me so much that I need personal help	3	0	1
In the school for the blind I am/was taught all skills to be able to act independently	1	2	1
Since I’m blind, I’m only in familiar places	2	0	2
Emotional	Yes	To Some Extent	No
The fact that I can’t see makes me:			
Sad	1	0	0
Angry	0	0	0
Worries	1	0	0
Afraid or anxious	1	0	0
I got used to it	3	1	0
I am afraid that as a blind person I will not be able to cope alone in the future	1	2	1
Social Environment	Yes	To Some Extent	No
I feel/felt accepted as I am	4	0	0
I’m afraid of making new contacts because of my blindness	0	0	4
I’m often asked about my appearance.	0	2	2
Other people don’t even notice the malformation of the eyes	1	2	1
The malformation of the eyes is a frequent topic of conversation	1	1	2
I have contact with other children who also wear one or two eye prostheses	2	1	1
Medical	Yes	To Some Extent	No
I have to see an ophthalmologist more than twice a year	1	1	2
I am afraid of visiting an ophthalmologist or ocularist	0	0	4
The prostheses often hurt	0	0	0
The prostheses sometimes hurt	0	0	0
The prostheses never hurt	4	0	0
The position of the prosthesis must be corrected more than once a year	2	0	2
I have other diseases	1	1	2

**Table 4:** Questions and answers of the parent questionnaires of the unilateral anophthalmos and microphthalmos patients (Uni) and bilaterally blind patients (Bi).

At the Time of Your Child’s Birth		Yes	To Some Extent	No
Already during the ultrasound examination during pregnancy, a possible physical change or malformation was detected in our child	Uni	0	1	14
	Bi	2	0	4
We were affected when we first saw our child at birth.	Uni	8	3	4
	Bi	3	2	1
The gynaecologist and the paediatrician were able to explain the change to us.	Uni	3	4	8
	Bi	1	3	2
We had to inform ourselves through books and the internet to understand what change it is.	Uni	8	5	2
	Bi	1	3	0
Treatment at the University Eye Hospital		Yes	To Some Extent	No
The gynaecologist and the paediatrician referred us to the Eye Clinic Rostock	Uni	2	2	10
	Bi	4	0	2
Only by own research we got to the Eye Clinic Rostock	Uni	9	3	3
	Bi	2	0	3
The team of doctors, nurses and ocularists answered our questions, fears and worries	Uni	12	1	2
	Bi	6	0	0
We were able to see the success of the operation after the first operation	Uni	11	2	2
	Bi	3	3	0
Costs of Treatments		Yes	To Some Extent	No
Our health insurance company of the statutory health insurance or private insurance company paid all costs such as travel, stays, operations, in full	Uni	7	5	3
	Bi	5	1	0
Our own costs accounted for more than 35% of the treatment costs	Uni	3	0	12
	Bi	0	2	4
There is concern about new costs in the course of further treatments	Uni	2	3	10
	Bi	2	0	4
Social Environment		Yes	To Some Extent	No
Our child gets/got along well in kindergarten	Uni	13	0	0
	Bi	3	2	1
Our child gets/got along well in the school (for the blind)	Uni	8	2	2
	Bi	3	2	1
Our child plays/played just like other children	Uni	7	2	6
	Bi	2	1	3
Our child is afraid to meet new children because of its own special appearance	Uni	0	5	9
	Bi	0	0	5
We are approached as parents about the appearance of our child.	Uni	4	7	3
	Bi	2	2	1
At first glance, others do not even notice the malformation.	Uni	3	7	5
	Bi	3	1	2
The malformation is a topic of conversation between us, the parents	Uni	6	5	3
	Bi	1	1	3
The malformation is a topic of conversation between us, the parents and our child	Uni	6	6	2
	Bi	3	0	2
The malformation is a topic of conversation between us, the parents and other parents	Uni	2	4	9
	Bi	2	2	2
The fact that our child can only see with one eye/is blind does not impact our social environment	Uni	11	2	2
	Bi	3	2	1
Our child has career plans	Uni	8	0	1
	Bi	1	1	4
We are in contact with other families and children in the same situation through a self-help group	Uni	0	1	13
	Bi	1	0	5
We are in private contact with other families and children who are in the same situation	Uni	3	1	11
	Bi	3	1	2

## Data Availability

The datasets used and/or analysed during the current study are available from the corresponding author upon reasonable request.
